# Public health: disconnections between policy, practice and research

**DOI:** 10.1186/1478-4505-8-37

**Published:** 2010-12-31

**Authors:** Maria WJ Jansen, Hans AM van Oers, Gerjo Kok, Nanne K de Vries

**Affiliations:** 1 Academic Collaborative Centre of Public Health Limburg, Maastricht, the Netherlands; 2 Caphri, School of Public Health and Primary Care, Maastricht University, Maastricht, the Netherlands; 3 Department of Public Health, National Institute of Public Health and the Environment, Bilthoven, the Netherlands; 4 Faculty of Psychology, Department of Work and Social Psychology, Maastricht University, Maastricht, the Netherlands; 5 Faculty of Health, Medicine and Life Sciences, Department of Health Education and Promotion, Maastricht University, Maastricht, the Netherlands

## Abstract

**Background:**

Public health includes policy, practice and research but to sufficiently connect academic research, practice and public health policy appears to be difficult. Collaboration between policy, practice and research is imperative to obtaining more solid evidence in public health. However, the three domains do not easily work together because they emanate from three more or less independent 'niches'.

Work cycles of each niche have the same successive steps: problem recognition, approach formulation, implementation, and evaluation, but are differently worked out. So far, the research has focused on agenda-setting which belongs to the first step, as expressed by Kingdon, and on the use of academic knowledge in policy makers' decision-making processes which belongs to the fourth step, as elaborated by Weiss. In addition, there are more steps in the policy-making process where exchange is needed.

**Method:**

A qualitative descriptive research was conducted by literature search. We analyzed the four steps of the policy, practice and research work cycles. Next, we interpreted the main conflicting aspects as disconnections for each step.

**Results:**

There are some conspicuous differences that strengthen the niche character of each domain and hamper integration and collaboration. Disconnections ranged from formulating priorities in problem statements to power roles, appraisal of evidence, work attitudes, work pace, transparency of goals, evaluation and continuation strategies and public accountability. Creating awareness of these disconnections may result in more compatibility between researchers, policy makers and practitioners.

**Conclusion:**

We provide an analysis that can be used by public health services-related researchers, practitioners and policy makers to be aware of the risk for disconnections. A synthesis of the social, practical and scientific relevance of public health problems should be the starting point for a dialogue that seeks to establish a joint approach. To overcome the above mentioned disconnections, face-to-face encounters consistently emerge as the most efficient way to transfer knowledge, achieve higher quality and acknowledge mutual dependence. We recommend practice and policy based research networks to establish strong links between researchers, policy makers and practitioners to improve public health.

## Introduction

Public health is the process of mobilizing and engaging local, regional, national and international resources to assure the conditions in which people can be healthy [1]. Public health includes three major fields: (i) policy, as it is inherently a political enterprise that supplies services and allocates resources; (ii) practice, as policies need to be implemented to create social action and organize service delivery; and (iii) research, as interventions need to be developed and assessed on effectiveness and cost-benefit ratios. A broad range of disciplines are relevant to these three major fields and public health as a whole. In fact, public health draws on biomedicine, epidemiology, biostatistics, genetics, nutrition, the behavioural sciences, health promotion, psychology, the social sciences (including social marketing), organizational development and public policy. These disciplines, each in their own way, have demonstrated that quality of life is a major topic in public health today. Ideally, policy, practice and research should be mutually dependent partners, uniting the different disciplines and combining academic and tacit knowledge to support public health. In reality, however, it appears to be difficult to sufficiently connect academic research, practice and public health policy. The three domains do not easily work together because they emanate from three more or less independent 'niches'. The term 'niche' is used here because policy, practice and research are characterized by specific ideologies as well as unique norms and values, internal orientations, communication and languages, internal codes of behavior, self-directed improvement processes, independence and a strong desire to protect themselves against the outside world [2-4]. Due to their niche character, the three domains do not easily converge, despite universal calls for collaboration [4-12]. Collaboration is thought to foster quality improvement of local and, ultimately, national public health policy in order to tackle complex public health problems. Quality improvement in the Dutch public health sector is urgently needed because, despite having boasted very good population health status in the past, the Netherlands, compared to the rest of the European Union, has seen a substantial decline in population health status in recent years. The assumption that collaboration between practice, research and policy will result in more solid evidence and higher quality standards in public health is widely supported [13-22]. Unfortunately, evidence does not naturally find its way into policy and practice [23].

Within the development of evidence-based medicine, a tradition of organizing practice-based research networks as a linkage between medical and public health practitioners and researchers has been built [24-28]. So far, these practice-based research networks mainly focus on the one-way transfer of evidence from research to clinical practice. However, gradually, more attention is being paid to the development of mutual relations that enhance practice-based knowledge production [29]. Public health can learn from these experiences of integrating research and practice. Within the field of public health, these practice-based research networks should be extended to include the policy domain. Although the policy domain is highly influenced by political aspects [30,31], policy-making should also be knowledge-based and result-oriented. This, however, poses several problems to the policy maker. The first problem is finding the evidence in the overwhelming volume of research literature. The second problem is the lack of monitoring and evaluation of public health policy that uses clear outcomes and performance indicators. The third problem is that the exchange process between policy makers, practitioners and researchers is often a one-way transfer. So far, the exchange has focused on agenda-setting or, as expressed by Kingdon [32], on how to create a window of opportunity. Next, it focused on the use of academic knowledge in policy makers' decision-making processes, as elaborated by Weiss [33]. However, in addition to agenda-setting and decision-making, there are more steps in the policy-making process where exchange is needed. These steps can be characterized as a regulatory policy work cycle [34,35]. The first step in policy is problem recognition, followed by an analysis of the problem and the formulation of an approach to solve it, which is step 2. Step 3 then involves the initiation of implementation and, lastly, in step 4, the effects are interpreted and evaluated. This stepwise procedure is based on the theoretical framework termed 'stages heuristic' or textbook approach [36] (p 6-7). Work cycles of practice and research have the same successive steps [37,38]. In reality, work cycles do not proceed rationally and linearly from step 1 to 4 [36]. Rather, they tend to be much more incremental [34,39] or a combination of both. Thus, work cycles proceed as a diffuse, open-ended, interactive and iterative process [36,39,40] which may make collaboration extremely complicated. Moreover, in order to include public health practice and research in the local or regional policy-making process, the benefits of such collaboration should fit closely with the goals and performance indicators of each domain.

In order to tackle these problems, reciprocity and interaction should be employed as the starting point for collaboration between legislative policy makers, practitioners and researchers [41-45]. Reciprocity and mutual engagement lack in most public health networks (if such a network, be it formal or informal, even exists). Although the framework of stages heuristic has outlived its usefulness, it is employed here as a means to better understand and unravel the extremely complicated collaboration process and to uncover risks for disconnections - or in niche terms - the different survival strategies in each step that keep the three niches separated. In an effort to promote improvements in the interaction between practitioners, policy makers and researchers, the awareness of these disconnections is considered to be the first step towards mutual understanding and initiatives for interaction and dialogue.

## Method

A literature search was conducted using (i) relevant textbooks; (ii) electronic bibliographic databases, namely Pubmed, Medline, Cochrane and PsycINFO; and (iii) reference lists of articles published in relevant journals. English and Dutch language articles and books published between 1980 and 2006 were included in the study. The search for relevant textbooks was based on more general terms such as structure, process and outcomes of research, practice and policy separately. The following key words were used for the electronic databases: public health collaboration, public health cooperation, inter-organizational relations in public health, inter-organizational exchange in public health, public health network, public health coalition, public health decision-making, evidence-based public health and practice-based evidence or policy-based evidence. In total, 204 references were found including textbooks or reports (n = 103) and articles (n = 101).

The work cycle model helped us to structure and interpret the literature. First, we explored the typical characteristics of the work processes in detail at each step. Generally used working methods were included in the work cycles. After this search was completed and the policy, practice and research work cycles were detailed for each step (Results part 1), comparisons were made between the steps of the three domains (subsequently for step 1 to 4) by describing the barriers for collaboration. The main conflicting aspects in each step were interpreted and confirmed by the literature in light of their impact on collaboration between policy, practice and research. This resulted in a descriptive overview of disconnections (Results part 2).

Given that, in most countries, the implementation of public health policy is the responsibility of the local authorities, the analysis presented here is from the perspective of local policy and local practice. Research can be conducted at any level and specification to local, regional or national level is indifferent in this analysis.

## Results

### Part 1: Work cycles

The constructed work cycles show double-sided arrows which represent the diffusion and iteration of the steps while words in bold represent the typical characteristics of the respective work cycles.

### The policy cycle

Policy is the process by which problems are conceptualized, solutions and alternatives are formulated, decisions are made, policy instruments are selected and programmes are delivered [34,35]. Public policy responds to social problems in order to solve, reduce or prevent them. Public problems can be solved by designing community actions [46-49], organizational actions, formal rules, procedures and routines [50-52]. The policy work cycle takes up to four years (generally the time that passes between elections). It is mainly carried out by civil servants and public administrators and is decided upon by the municipal or city council. Civil servants are rewarded when they operate without failure, which may result in risk avoidance and routine behavior [30,31,35]. Public administrators and decision makers like aldermen and politicians who participate in the city council often make policy choices on the basis of how those decisions will impact their chances of being re-elected (i.e. popular and visible) [30,31,35,53]. They want to survive in their niche.

#### Step 1: Recognition of a **socially relevant **problem

**Figure 1:**
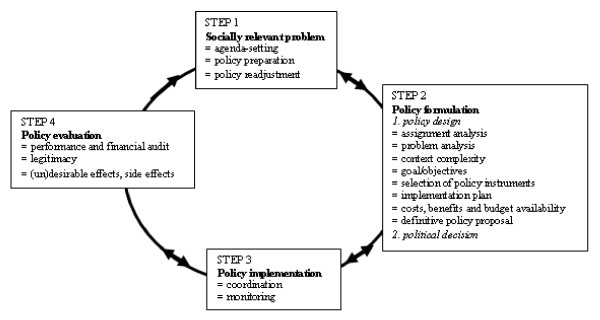
**The regulatory policy cycle **[42].

A problem is described as a discrepancy between negotiated and democratically defined basic social principles and the current situation [30,32]. The perception of problems therefore depends on a comparison between current normative standards and the actual situation. Normative standards are influenced by the ruling political parties and by public common sense. **Problem recognition **is often interpreted as mainly a matter of strategic representation of the situation [30]. In the eyes of the public, this may be regarded as window dressing or following the hype of the day. **Agenda-setting **is a crucial aspect of step 1. How issues are placed on the policy agenda or how they may be prevented from being placed there is a complex process, which is often highly unpredictable [32]. Important criteria for agenda-setting are whether the involvement of the government is legitimate and the policy instruments are accepted by the prevailing political ideology and the majority of the population and whether political salience, public visibility and personal immediacy are positively valued [13,54-56]. When a topic has been put on the policy agenda, the alderman can for instance appoint a sectoral or intersectoral committee for **policy preparation**, i.e. the general framework for elaborating on the topic. During the subsequent stage of policy formulation or implementation, a **policy readjustment **can be considered necessary because of, for instance, negative mass media, unfeasibility or undesirable side effects [35,53]. Figure 1. The regulatory policy cycle [42]

#### Step 2: Policy formulation and political decision

The formulation of policy starts with an analysis of the policy domains that have to be involved, i.e. the **assignment analysis**. One has to decide whether a problem belongs to, for example, the education, public housing or public health department, or to all policy departments. Departments have their own legally defined authority status and informal power. For instance, the department of city planning often has more power, both formally and informally, than the departments of social welfare and public health [30,31].

The **problem analysis **includes many different perspectives, i.e. from the perspective of (population) health, economics, socio-economic differences, employment, social participation, spatial planning, etc., and the same holds for the proposed problem-solving strategies. Public administrators take the **context of societal complexity **as a starting point for policy formulation [57]. **Goals and objectives **are formulated in general terms. The **policy instruments **are then selected and may include information, community action, economic incentives (e.g. subsidies), legal directives and organizational arrangements [34,35,47,52]. Then an **implementation plan **is developed which leads to negotiations with different local organizations in order to make agreements on their contribution. A specification of **costs and benefits **must be completed and **budget availability **must be explored before the **definitive policy proposal **can be submitted to the municipal council. The **political decision**-making process can be characterized as a process of bargaining, lobbying, negotiating and accommodating different interests [30,31,53,58]. Final decisions are often the result of compromise.

#### Step 3: Policy implementation

The implementation process is often insufficiently **monitored **[42,53]. In many cases, there is no clear 'road map' showing what, where, when, how and by whom activities are to be implemented. Many policies are described in terms of policy intentions instead of SMART-formulated policy goals (Specific, Measurable, Acceptable, Realistic and with Time specification) which makes monitoring rather difficult [30,59-61]. Despite the fact that local government claims to be in charge of local public health, the **coordination **and the role of the process manager often is unsatisfactorily implemented, especially when a range of partners from different sectors are involved [62,63]. Task allocation, responsibilities and competences are often left undefined [64,65].

#### Step 4: Policy evaluation

Policy evaluation is often considered unimportant. At any rate, many policy programs are not evaluated at all [66,67]. In some countries, the local government is obliged to audit its performance. Audits can be interpreted as an evaluation method. **Performance audits **and **financial audits **seek to verify the degree to which policy conforms with pre-defined performance indicators or budgets, respectively. When there is a formal duty for public accountability for the estimation of the impact of public health policy budgets on the health of the public, like in the Netherlands [68], still then its fulfillment is often weak [61,68-71]. As ambiguous goal formulation makes effect evaluations that are based on academic standards rather difficult, it may only be feasible to evaluate in terms of process criteria or intermediate goals [30,61,64,72,73]. Evaluation research determines **desirable and undesirable effects **and **side effects**, and - important for public policy - **legitimacy**. These aspects play a rather important role in generating accountability for civil servants' political responsibilities [30,31,34,59,74]. Legitimacy during the formulation phase can be misjudged, can change over time or can be evaluated differently because of a change in the ruling political party. Failing public acceptance of government interference and acceptance of the policy instruments selected, a policy will not likely be continued. Although many evaluation results contribute to the knowledge and expertise of the public administration, there are many cases in which evaluation results are deliberately not published or communicated as this might be too risky to the political elite [30,31].

### The practice cycle

Practice aims to serve the needs of others, either directly or indirectly. Practitioners in public health primarily want to solve problems immediately and meet the needs and demands of their clients with whom they have a direct, personal contact. Practice has a preference for a short cycle because practitioners feel a sense of urgency [75]. In this way they can survive within their own niche.

#### Step 1: Description of a **practically relevant **problem

A problem is defined as a discrepancy between the actual situation and the **needs and demands **perceived by individuals, groups, communities and local authorities. A problem is perceived when the current normative standards do not correspond with the actual situation as expressed by epidemiological findings, political priorities and public demands [55,75,76]. To recognize a problem, practitioners must perceive a difference between what currently exists in their consultation room or community and a more desirable state which they believe is attainable, modifiable and tractable [75]. Problems in practice are concrete and detailed, and practitioners focus on ways to act immediately, rather than on ways to reason, generalize or find the evidence as researchers do, or wait for legitimate policy instruments and finances as policy makers do.

**Figure 2:**
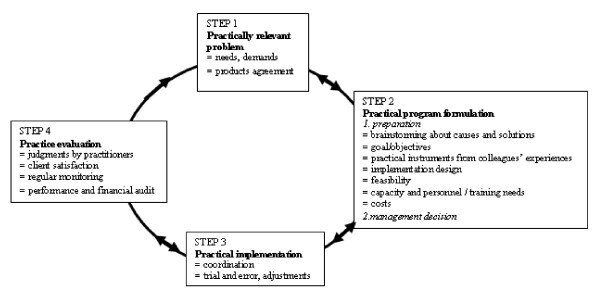
**The regulatory practice cycle **[42].

In many countries, practitioners' routine work is based on the **product agreements **between local authorities and their Public Health Services. The management of Public Health Services can only invest in the development of new programs to solve identified problems when this is permitted by the budget and when capacity is sufficient. Otherwise, negotiations with local authorities have to be initiated to increase budgets. Figure 2. The regulatory practice cycle [42]

#### Step 2: Practical program formulation

Practitioners almost always experience time constraints due to organizational and personal factors. The practice domain always has an organizational duty to deliver products fixed in scope and frequency as determined each year by the local government. Practitioners do not have time for exhaustive behavioral, environmental and educational analyses [76,77] or for acquiring profound theoretical insights for the selection of practical strategies [75]. Because practitioners assign high intellectual status to scientific research they do not easily contact researchers for support. **Brainstorming about causes and solutions **and adopting ready-to-use **practical strategies **from **colleagues' previous experiences **are fairly quick and easily accessible procedures [78], which are often applied without a systematic validation of the context [79].

**Goals and objectives **are not often specified in details [75,80] and the **implementation design **usually pays significant attention to organizational constraints and the practical benefits. Practitioners' attitudes make them creative in terms of solving **feasibility **problems. **Capacity, personnel and training needs **are taken into account, resulting in the total **costs **[80]. Agreement on the design is required by management. Local authorities have to make decisions when financial support is necessary or when the program poses future budgetary risks. This can result in **management decisions **being delayed for a couple of months while practitioners tend to be in a hurry. Practitioners often insufficiently anticipate the policy developments required to integrate program activities in local policy [42].

#### Step 3: Practical implementation

If practitioners design a program in close cooperation with all colleagues who have to use it, implementation is usually not perceived as a problem [77]. **Coordination **agreements are made and the program can be delivered by local and/or national organizations. Incompletely worked out designs are improved by **trial and error **sometimes resulting in 'muddling through' [35].

#### Step 4: Practice evaluation

The final step of the cycle is a frequently neglected aspect in public health practice as it requires both a theoretical and practical attitude. Evaluation tends to be at the bottom of the practitioners' list of priorities and budget items [66]. At best, the evaluation consists of **practitioners' judgments **of the program delivery and an assessment of **client satisfaction **as part of routine quality improvement procedures. Practitioners' work is not paid on the basis of health outcomes at the individual or population level but rather on the basis of whether the product agreements are met. Public Health Services are obliged to produce an annual report on their **performance and financial auditing **[68,81]. This planning and control cycle, however, functions as a productivity report rather than an evaluation of public health. Outcome data, documented in terms of life expectancy, prevalence of diseases or public health problems, are available from the **regular monitoring **services but are not linked to the auditing process. Consequently, the effect of a program on public health practice over time cannot be specified. Such an effect evaluation is also difficult in light of the general constraints and difficulties associated with measuring the effects of preventive public health services [82-84].

### The research cycle

Scientific research is defined as the systematic, controlled, empirical and critical investigation of hypothetical propositions about presumed relations among natural phenomena. Scientific research aims to produce explanations and predictions - and in case of the applied sciences, also solutions - relating to people's problems, and to contribute facts and theories to the body of knowledge [85]. The scientific approach is the most systematized method of acquiring knowledge. This orderly pattern is called the empirical cycle [37,86]. The empirical cycle refers to the process in which evidence rooted in objective reality (assuming that an objective reality exists independent of human discovery or observation) and gathered through the human senses is used as the basis for generating knowledge.

The research cycle takes about four years or more as many research projects, e.g. PhD dissertation projects usually take four years, but eight to ten years may pass from the time of the initial hypothesis or research question to publication and dissemination [29]. The way the researchers work helps them to survive in their niche

#### Step 1: Defining a **scientifically relevant **problem

**Figure 3:**
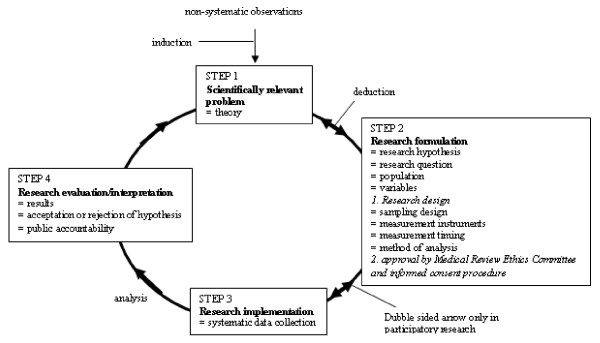
**The empirical research cycle **[42].

In the research domain, a problem is described as a discrepancy between theory and reality, between different theories, between theory and practice, or between practice and desired practice [86,87]. A problem is perceived as scientifically relevant when, by systematic empirical observation, information can be accumulated or theories can be formulated to extend the existing knowledge base. Step 1 is the generalization of non-systematic observations or perceived practical problems to a problem that is based on **theory **[59]. Scientifically relevant problems originate from passionate researchers who integrate observations in a more abstract, generally valid picture of reality through creativeness, imagination and induction. Figure 3. The empirical research cycle [42]

#### Step 2: Formulation of research design and hypothesis

A **hypothesis **formulated via deductive reasoning is a tentative prediction or explanation of the relationship between two or more variables [37,59,87]. The hypothesis serves as a link between the theory and the real-life situation. Descriptive, exploratory and phenomenological studies may not require a hypothesis beforehand as their aim may be to develop hypotheses. The **research question**(s) and the research design are thoroughly elaborated, which is generally a time-consuming process. The **population **to be studied and the **variables **involved are delimited. Researchers tend to reduce complex problems to a range of more detailed problems that can be studied separately. Researchers need to be experts in developing the **research design**, **sampling design**, **instruments to measure **variables, **timing **and **methods of analysis **[37,59,85,87].

In public health research, humans are often the source of information. Great care must be exercised so that the rights of these humans remain protected. A **medical review ethics committee **must approve the study and the procedure for obtaining **informed consent **needs to be addressed [88].

#### Step 3: Research implementation

In many studies, the empirical phase, i.e. the **systematic data collection**, is the most time-consuming part of the investigation. Researchers do not always sufficiently anticipate problems relating to practical matters like data collection or registration systems, the controlled application and fidelity of the intervention in practice [17,20,81,86], logistic requirements, the identification of partners who need to be involved or committed, the involvement of qualified staff, research design soundness guarantees during implementation, recruitment procedures and resource availability.

#### Step 4: Research evaluation

Although **hypotheses can be accepted or rejected**, it is inappropriate to speak of definite proof because this is incongruent with the limitations of the scientific approach. Scientists constantly seek objective, replicable evidence as the basis for understanding phenomena. The more frequently the same **results **are found, the greater the confidence in their validity [89].

Scientific researchers have a duty in terms of **public accountability **[86] and should communicate their findings to an audience. Four types of audiences are distinguished: the scientific forum, the institutions funding the research project, the practice forum and the general public. Accountability to the scientific forum has a twofold function, i.e. to assess whether the results and the design can stand the test of scientific criticism and to contribute to the scientific body of knowledge. The productivity of a researcher is often assessed by the number of scientific publications one has in influential international journals with a high citation score [90]. Because of this, publications in professional journals specifically meant for the practice forum are less valued thus impeding the dissemination of findings to practice. Scientists are expected to keep a certain distance from policy or practice, avoiding public controversy [91] and emphasizing their objectivity and neutrality [92].

### Part 2: Disconnections

Table 1 summarizes the differences between the niches for each step in the work cycle (steps as labels and differences as sub-labels) that may result in disconnections. The niche is an ecological term. Several species can populate the same, different or overlapping niches. For each step, we show why the human species of policymakers, practitioners and researchers have populated different niches to maintain a stable and livable group. Next, we suggest the required burden of tolerance that is needed to cohabit with other species in overlapping niches.

**Table 1:** Differences (in italics) between the work cycles in the three niches.

	Policy	Practice	Research
**STEP 1** **Problem recognition**			

**1. Relevance**	*socially relevant *problem, i.e., solving social problems, influenced by political parties	*practically relevant *problem, i.e. corresponding to the public's or client's requests or needs due to problems that are modifiable and tractable	*scientifically relevant *problem, i.e. explaining problems and adding to the body of knowledge based on existing theory

**2. Policy agenda setting**	*much influence *on agenda setting	*limited influence *on agenda setting, media pressure	*very limited influence *on agenda setting

**3. Status**	*bureaucratic *status	*social *status	*high intellectual *status

**STEP 2** **Formulation of policy, practice and research**			

**4. Formal power in policy**	*much influence *of small political group on policy formulation	*sometimes indirect influence *on policy formulation	*usually no influence *on policy formulation

**5. Goals**	*Insufficient transparency *of final goals	*limited transparency *of final goals, restricted to practice	*sufficient transparency *of final goals, but restricted to research

**6. Evidence**	*policy-based evidence*: legitimacy, acceptability, visibility, immediacy, political salience	*practice-based evidence*: profitability, applicability, feasibility	*research-based evidence*: rationality, empirical validity, logical precision

**7. Legitimacy**	*preferred focus on environmental *approach, social, physical, economic	*focus on individual *behavioural approach	*insufficient focus on environmental *approach

**8. Value of theory and practice**	theories are *partly relevant*; practical implementation is *relevant*	theories are *irrelevant*; practical implementation is *relevant*	theories are *relevant*; practical implementation is often *irrelevant*

**9. Work attitude**	work attitude of *administrative control *and opportunism; some creativity involved	firm, *action-directed *work attitude; 'quick and dirty'; creativity involved	*cautious *work attitude; detailed and time consuming; creativity involved

**STEP 3** **Implementation of policy, practice and research**			

**10. Adjustments during pilot**	*interim policy adjustments *during policy pilot, trial and error approach	*interim practical adjustments *during pilot, trial and error approach	*no interim adjustments*, except for qualitative, responsive research

**STEP 4** **Policy and practice evaluation and research interpretation**			

**11. Lifespan**	*unpredictable lifespan *of work cycle, maximum four years	*preferably short lifespan *of work cycle	*predictable lifespan*, depending on research design and public availability, 4 to 10 years

**12. External vs. internal validity**	*need for external validity *but policy results often too tentative	*need for external validity *but practical implementation and contextual factors often undefined	*focus on internal validity*, insight in what is effective but not in how it can be made effective in real world setting

**13. Public accountability**	*increasing public accountability*, mainly financial within own field	*limited public accountability*; mainly financial within own field	*public accountability *by publications in highly authoritative journals within own field

In **step 1 **policy makers (legislators) define a public health problem in terms of its **relevance **to their political ideology and public opinion [15,30-32,53,93]; practitioners define the same public health problem in terms of its relevance to perceived needs and demands of individuals, epidemiological findings, and products agreements [75,80]; and researchers define the same public health problem in terms of its relevance to theory, existing body of knowledge, and interests of the investigator [59,81,87]. The starting points are different as social, practical and scientific relevance do not automatically overlap [4,14,15,30,32,34,92,94,95], but species do not exploit each other and can search for a new equilibrium. Besides, the decision to start the policy cycle is made by a small number of city councillors who together decide to put a subject on the policy agenda [32]. Practitioners and researchers have no formal authority in local policy **agenda setting **and cannot easily influence local policy, although they can use media attention to put a topic on the political agenda. Nonetheless, most of the time, each field of policy, practice and research sets its own agenda thus leaving the gap between the fields as it is [42,96].

Each field is valued differently by the other fields in terms of **status**. Policy makers, and even more so practitioners, assign high intellectual status to scientific research. They place research at a distance and do not value research extensively because of its high intellectual requirements [42]. Scientists and practitioners, on the other hand, perceive the policy making process as highly bureaucratic, impenetrable and full of delays [30,31,53,58]. Scientists generally perceive practice as socially relevant, but they are not always interested in the 'real world', thereby implicitly and unconsciously lowering social status of practitioners [43,92].

In **step 2 **practice and research have no **formal decisionary power **over policy formulation and they have limited influence on the political decision to agree or not on a policy proposal [32]. The final policy **goals **of these proposals are often expressed in policy intentions that are hard to measure [30,31,34,66]. The same holds for the practice goals. Research goals, on the other hand, are expressed in detail and in SMART terms [37,87].

**Evidence **[97] has different meanings in each cycle. The term 'evidence-based' is principally based on rationality but other interpretations of the term evidence have developed [18]. Essentially, these reflect the viewpoints of the parties concerned, as can be seen with the terms 'practice-based evidence' and 'policy-based' or 'policy-informed evidence' [7,81,98-103]. The terms 'policy-based evidence' and 'practice-based evidence' contribute aspects that originate from their respective niche characteristics. This means that, whereas rationality, empirical validity and logical precision are the decisive arguments for researchers thus resulting in the concept of research-based evidence, legitimacy, public acceptability, political salience, public visibility and public immediacy are the added decisive arguments for policy makers to act or refrain from action, and these arguments shape the concept of policy-based evidence [96,98,99,101,104]. From the practitioners' perspective, meeting the needs of individuals and groups as well as feasibility, profitability and applicability are the added decisive arguments to act [75]. These are expressed in the concept of practice-based evidence.

**Legitimacy **is an important aspect during the process of policy formulation [31]. Last decades, public health practice and research have tended to focus on individualized approaches to risk management [58,105,106]. However, policy actions focusing on behavioral lifestyle determinants are considered moralistic and may be politically controversial because they interfere in people's private lives [58,107]. The environmental determinants have been unsatisfactory investigated so far to formulate effective public health policy. If the legitimate role of policy is to be linked to research and practice, the environmental dimension of health should be more explicitly defined in research and practice [58,108-110].

The role and value of **theory and practice **are different in each niche. Theory is the starting point or the final goal of research and is regarded as indispensable [59,89]. Within policy-making, the use of theory depends on the educational background and academic experience of civil servants [111]. Practitioners do not tend to use theories to explain how they expect their activities to work. Theories consist of impractical, high-flown, unrealistic ideas, are abstract and are used when there are no facts [95]. Researchers, on the other hand, tend to find practice-based knowledge scientifically irrelevant.

Policy makers, practitioners and researchers have a different **work attitude**. Scientists are regarded as thinkers, practitioners as doers and policy makers as bureaucrats [30,31,34,42]. These stereotypical images hamper collaboration as they can become ingrained prejudices. Although research findings are often regarded as tentative by scientists, practitioners expect to receive clear guidance on how to act. A cautious scientific attitude may thus clash with a firm attitude towards action. Practitioners may feel inhibited while researchers must fight for the time-consuming accuracy they strive for. The administrative function of the authorities often results in a controlling, bureaucratic and opportunistic attitude, which may conflict with the creative thinking and actions of researchers and practitioners [41,42]. To cohabit with other species in overlapping niches requires acceptance of differences in power and working style, and training in other languages to understand evidence, legitimacy and the dichotomy of theory and practice.

In **step 3 **the problem of **interim adjustments **may appear. Whereas adjustments during implementation are strongly discouraged in most research designs - except for qualitative and responsive research methods -, they are acceptable in policy and practice. As policy- and practice-related knowledge advances during the implementation stage, it influences the formulation of policy and practice programmes and readjustments are made. Repeated switches from step 3 to 2 and vice versa is called 'muddling through', which is not allowed within the field of research, unless it concerns participatory action research. Once a research design has been selected and interventions have been defined, readjustments to the intervention are not allowed anymore [37,87]. This kind of control will sometimes demand huge sacrifices and inflexibility from the practice field which may even be confronted with client dissatisfaction [107]. When researchers and practitioners have not sufficiently anticipated problems relating to practical data collection or registration systems, controlled application and fidelity to the intervention in practice, logistic requirements, identification of partners who need to be involved or committed, qualified staff, recruitment procedures and resource availability, the research conduct might get stuck [17,20,112]. In niche terms, interim adjustments can be considered a predator that should be made innocuous.

In **step 4 **the results have to be interpreted. Each cycle has its own dynamics and **lifespan**. Research and policy projects usually take four years while practical programmes have a short lifespan. Unforeseen arguments within the political arena sometimes cause cycles to start during the period in between elections, and their duration can then hardly be predicted [32]. This combination of different paces and the desired interconnections between the cycles makes meshing extremely complicated.

After a research project has been ended, researchers no longer have a legitimate role in the translation of results into policy or practice. As research tends to reduce the complexity of real life problems to detailed sub-questions that are studied separately, it is often difficult to offer an integrated problem solution, ready-to-use in practice or policy [113]. Researchers pay more attention to the **internal validity than to the external validity**, i.e. the generalization of the results [104,114]. Action on any substantial scale often has to wait for further analyses that address the contextual determinants in order to corroborate the evidence in practice or policy.

Policy makers, practitioners and researchers each have a duty of **public accountability**, but in different ways [68,86]. Audit reports of practice and policy, and peer reviewed, scientific journals are, in theory, accessible and thus readable to the general public, but access is hampered by a range of barriers relating to organisational structure. Besides, the content of peer reviewed, scientific articles is not readable for politicians, civil servants or public administrators due to its scientific jargon, and if it is readable for practitioners they often lack time. The other way round, the content of memoranda from local government or public health service is not attractive to read for researchers due to its length, lack of new knowledge and ambiguous formulation which is necessary to serve consensus and cooperation. To summarise, all kinds of publications are nearly exclusively used within the individual fields that produce them. When species connect different timelines and assist each other in generalizations and professional and scientific publications, the species can live together in overlapping niches.

## Discussion

This review of the three work cycles and description of current public health policy, practice and research shows that there are some conspicuous disconnections that strengthen the niche character of each domain and hamper integration and collaboration. Improving collaboration between the public health niches and their work cycles requires, first and foremost, awareness of these differences. Mutual understanding may subsequently reinforce mutual respect and collaboration. As each work cycle starts with the recognition of a problem, respective professionals need to achieve a synthesis with respect to the social, practical and scientific relevance of public health problems. Priorities regarding agenda-setting, problem formulation, goal clarity, evidence use, legitimacy, theory use, attention to internal and external validity, lifespan and the availability and readability of publications do not automatically overlap. Formal power and status differ between the three niches. Kingdon and Weiss described barriers to exchange between policy, practice and research during agenda-setting and decision-making.

We add to that body of knowledge the barriers in all steps in the exchange process. Given the thirteen disconnections, we contend that meshing the desired interconnections between the cycles is an extremely complicated endeavor [41,42].

## Conclusion

To overcome the above mentioned disconnections, face-to-face encounters consistently emerge as the most efficient way to transfer knowledge, achieve higher quality and acknowledge mutual dependence [94,113,115,116]. Personal relations provide gateways to the knowledge available in other niches and may result in affective ties that subsequently can reduce status differences. These can, in turn, stimulate receptivity and commitment to the other niches. Professionals are thus given access to the internal structures of other niches, their formal and informal networks and their climate and culture, which can help them to cross niche barriers and speed up intersectoral knowledge circulation. Public health policy, practice and research must work in consort in each step of the work cycle [92,117,118].

Furthermore, managers of practice institutions and public health professors should endeavor to get involved in the political elite as social entrepreneurs [32] and this may enable them to exert effective influence on agenda-setting, policy formulation and political decision-making. News media publications rather than scientific publications in influential international journals are needed to address the public in general and politicians in particular. Thinking in terms of a theory-practice continuum or a synthesis will also promote knowledge about public health evidence. The challenge is to find performance indicators that yield mutual benefits because collaboration does not start or continue automatically. Each niche has arguments that can be used to defend their actions and collaboration should combine the best of each approach in an effort to achieve added value and quality improvement in public health.

Our findings suggest the need for novel structures that bridge policy, practice and research. In 2005, nine centers for collaboration between public health policy, practice and research were initiated in the Netherlands [119,120]. These centers are called 'Academic Collaborative Centres for Public Health' (In Dutch, *Academische Werkplaats*). These centres create one biotope in which three niches, each with their own burden of tolerance, can live together because no mutual exploitation mechanisms exist. Hopefully, such a biotope can teach us important lessons regarding this transformative process, which, in turn, will add to the knowledge we have thus far.

## Competing interests

The authors declare that they have no competing interests.

## Authors' contributions

MJ contributed to the design and conduct of the study and interpretation of the results and drafted the manuscript. HO, GK and NV helped critically revise the manuscript in terms of content. All authors read and approved the final manuscript.
